# Engineered Mesoporous Silica-Based Core-Shell Nanoarchitectures for Synergistic Chemo-Photodynamic Therapies

**DOI:** 10.3390/ijms231911604

**Published:** 2022-10-01

**Authors:** Yue-Mei Gao, Shih-Han Chiu, Prabhakar Busa, Chen-Lun Liu, Ranjith Kumar Kankala, Chia-Hung Lee

**Affiliations:** 1 Department of Life Science, National Dong Hwa University, Hualien 97401, Taiwan; 2 College of Chemical Engineering, Huaqiao University, Xiamen 361021, China

**Keywords:** mesoporous silica nanoparticles, core-shell nanoarchitectures, photodynamic therapy, chemodynamic therapy

## Abstract

Combinatorial therapies have garnered enormous interest from researchers in efficiently devastating malignant tumors through synergistic effects. To explore the combinatorial approach, multiple therapeutic agents are typically loaded in the delivery vehicles, controlling their release profiles and executing subsequent therapeutic purposes. Herein, we report the fabrication of core (silica)-shell (mesoporous silica nanoparticles, MSNs) architectures to deliver methylene blue (MB) and cupric doxorubicin (Dox) as model drugs for synergistic photodynamic therapy (PDT), chemotherapy, and chemodynamic therapy (CDT). MB, as the photosensitizer, is initially loaded and stabilized in the silica core for efficient singlet oxygen generation under light irradiation towards PDT. The most outside shell with imidazole silane-modified MSNs is immobilized with a chemotherapeutic agent of Dox molecules through the metal (Copper, Cu)-ligand coordination interactions, achieving the pH-sensitive release and triggering the production of intracellular hydrogen peroxide and subsequent Fenton-like reaction-assisted Cu-catalyzed free radicals for CDT. Further, the designed architectures are systematically characterized using various physicochemical characterization techniques and demonstrate the potent anti-cancer efficacy against skin melanoma. Together our results demonstrated that the MSNs-based core-shell nanoarchitectures have great potential as an effective strategy in synergistically ablating cancer through chemo-, chemodynamic, and photodynamic therapies.

## 1. Introduction

Cancer has emerged as one of the leading reasons of death globally, resulting in millions of deaths annually [1,2]. Over the past decades, several efforts have resulted in understanding the origination, etiology, and development of cancer, resulting in the development of advanced approaches for its early detection and diagnosis [3,4]. Over the past few decades, various therapeutic strategies, such as surgical procedures, radiation, and chemotherapy, have been practiced in ablating cancer [5,6]. In recent times, nanoparticle-based delivery vehicles have become a rapidly growing area in the biomedical field due to their site-specific delivery [7,8], physiological stimulus delivery [9,10], and fewer adverse effects.

Reactive oxygen species (ROS) play an important role in regulating cellular integrity [11,12]. For instance, ROS can be endogenously generated from the NADPH enzyme [13,14] and mitochondrial electron transport chain [15,16]. In cancer therapy, the intracellular generation of excess ROS can provide ablating effects, decreasing cell survival and inhibiting the proliferation of cancer cells [17,18,19]. Since cancer cells can produce a higher hydrogen peroxide concentration than normal cells, the intracellular provision of metal ions often facilitates ROS production through various catalytic reactions. Typically, various kinds of toxic ROS, including hydroxyl radicals (**.**OH), superoxide anions (•O_2_^−^), and singlet oxygen (^1^O_2_), can be exogenously produced by photodynamic [20,21,22], chemodynamic actions, and Fenton-like reactions [23,24,25]. The imbalance in ROS levels could generate oxidative damages, such as lipid peroxidation, DNA fragmentation, and protein denaturation, triggering cell apoptosis and necrosis [26].

In recent years, the combination of chemo- and photodynamic therapy (PDT), referred to as a synergistic therapeutic strategy, has emerged as the most promising therapeutic strategy in significantly ablating cancer cells. More often, these synergistic therapeutic strategies are operated by administrating specific chemo-drugs and photosensitizers that are applicable to localize at the desired site and concomitantly be activated with light to execute their therapeutic effects [27,28,29]. Despite the advantages and success in killing cancer cells, these photosensitizers for PDT suffer from limitations such as low solubility, poor cellular uptake efficiency, and stability under physiological conditions, subsequently leading to low efficiency. During chemo-photodynamic therapy, several predominant issues must be considered, such as improving their stability and bioavailability in the target cells. Recently, Dong et al. exploited metal- and metal-oxide-based nanoparticles for cancer PDT. Using cerium-based nanoparticles as novel photosensitizers presented an efficient quantum yield for ^1^O_2_ generation and avoided photobleaching effects under light irradiation [30]. In another case, Zhang et al. reported the synthesis of FePS_3_-PEG nanosheets, which showed photothermal effects with good biocompatibility for excellent therapy effects under near-infrared irradiation [31].

Methylene blue (MB), a phenothiazinium photosensitizer, has attracted great interest from researchers in its applications in biomedicine, specifically PDT, due to its high quantum yield of singlet oxygen generation, applicable therapeutic window, and low dark toxicity. However, MB shows the drawbacks to PDT-associated limitations, such as poor stability for photo (or enzyme) degradation and low cell membrane transport. To overcome these limitations, several efforts have been dedicated to developing various inorganic nanoparticles and polymer-based nanocarriers [32,33].

Among various nanocarriers, mesoporous silica-based nanocarriers (MSNs) have gathered massive interest from researchers to deliver various photosensitizers and chemo-drugs due to extensive surface area, high biocompatibility, tunable particle sizes, ease of controlling the functional group distribution, and excellent biocompatibility, among others [34,35,36,37]. MSNs have been extensively applied as a carrier in biomedicine and pharmaceutical research, such as imaging [38,39,40], targeted drug delivery [8,41,42], and gene delivery [43,44], among others [34,35,45]. For instance, Santi et al. synthesized mesoporous silica-loaded MB with amino and mannose-targeted nanoparticles to treat the multi-drug resistance microorganism [46]. Yunhong Yi et al. synthesized plasmonic nanoparticle MB-loaded gold nanobipyramids@SiO_2_ to generate singlet oxygen ^1^O_2_ to treat cancer cells [47].

**Figure 1:**
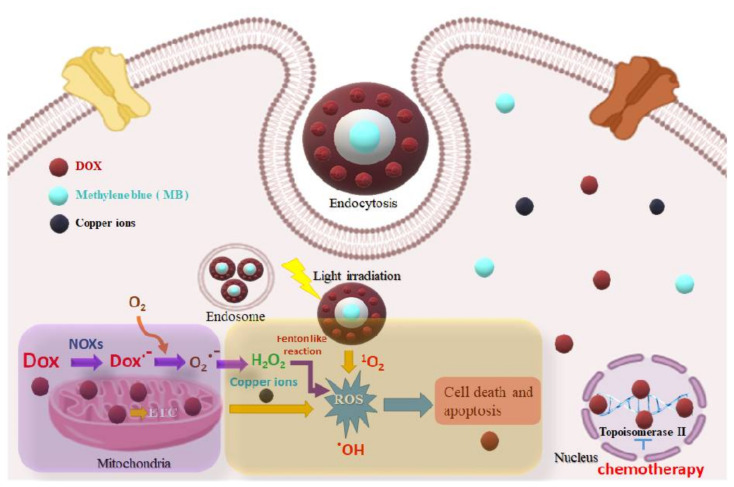
Schematic representing the plausible mechanisms of the core-shell nanocomposites for chemo-, chemodynamic, and photodynamic therapies.

Inspired by these facts, we fabricated MB-SiO_2_@MSNs core-shell nanocomposites, considering the photosensitizer MB in the silica frameworks as a core, which was coated with imidazole-modified mesoporous silica as a shell to immobilize cupric ion and doxorubicin (Dox) for controlled release through metal coordination for the synergistic effects of chemo-, chemodynamic, and photodynamic therapies (Dox-chemotherapy, cupric ions-CDT, and MB-PDT) to cancer therapeutics (Figure 1). These core-shell architectures were designed to facilitate the embedded MB in the silica frameworks with easy energy transfer to oxygen via mesopores for PDT-based activation to singlet oxygen in ablating cancer cells. Furthermore, the chemotherapeutic drug (Dox) had a high loading rate into the mesopores via metal–ligand affinity interactions from the pH-sensitive copper (Cu)-Dox coordination bond. Dox can additionally induce the production of intracellular hydrogen peroxide in cancer cells; therefore, Cu metal ions in the mesopores synergistically enhance the ROS levels by catalytic cycles through the Fenton-like reactions.

## 2. Results and Discussion

**Figure 2:**
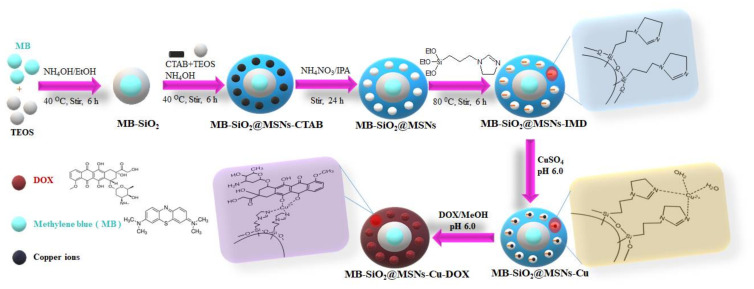
Schematic representation showing the synthesis of MB core-shell nanoparticles with surface modification of imidazole group for metal coordination and further immobilization of Dox.

As shown in the schematic illustration (Figure 2), this work accomplishes all the following aspects. Initially, we prepared MB-SiO_2_ nanoparticles, in which the photosensitizer MB molecules were monodispersed in the silica framework to avoid aggregation. In addition, the negative charges of the silica skeleton form strong electrostatic interactions with MB to further improve the stability of the photosensitizer and the energy transfer efficiency of PDT. Further, the growth of mesoporous silica shells on the MB-SiO_2_ can increase the stability of MB molecules to prevent their leaching during the delivery processes. The MB-SiO_2_@MSNs showed fine spherical morphological structures of core-shell nanoparticles. After removal of CTA^+^ by isopropanol-based chemical extraction, mesopores were modified with imidazole silane for Cu ion chelation. The use of metal affinity interactions can achieve the high loading of Dox along with pH-sensitive controllable release to enhance their efficiency for chemo-photodynamic combination therapy towards melanoma cancer cells.

### 2.1. Preparation and Characterization of MB-SiO_2_@MSNs

**Figure 3:**
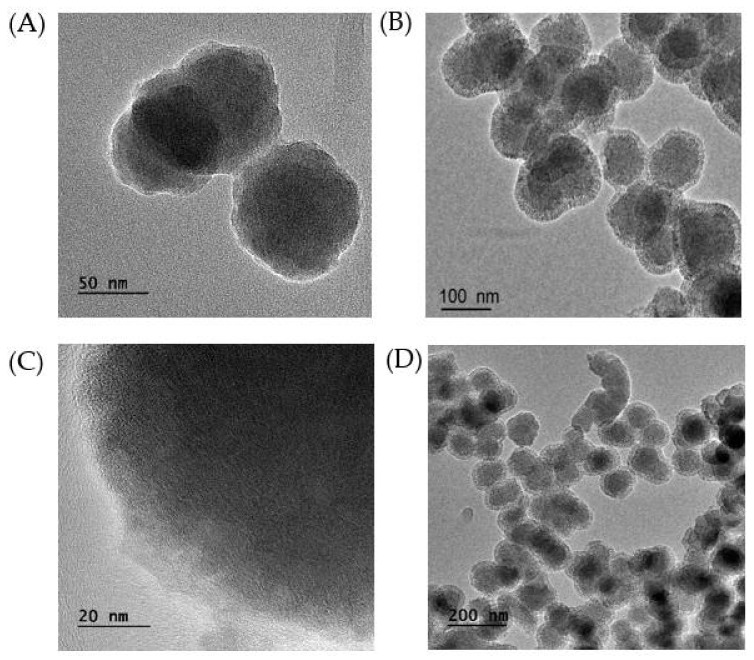
TEM images of (**A**) MB-SiO_2_ and (**B**–**D**) MB-SiO_2_@MSNs nanocomposites.

The TEM image of MB-SiO_2_ nanoparticles (Figure 3A) revealed that the MB-SiO_2_ NPs showed a spherical shape with an average diameter of 50 nm. The spherical MB-SiO_2_ with a highly dense core facilitated the successful encapsulation of MB in the silica frameworks with tunable morphology and size. Further, mesoporous silica was grown on the surface of MB-SiO_2_ by the sol-gel co-condensation method. The results showed that MSN shells with the proper thickness (20 nm) and porous structures existed in the outside shells of the nanocomposites. Together, the overall morphology of the designed MB-SiO_2_@MSNs core-shell nanoarchitectures was a spherical and uniform diameter of around 200 nm, with a uniform mesoporous shell (Figure 3B–D). 

**Figure 4:**
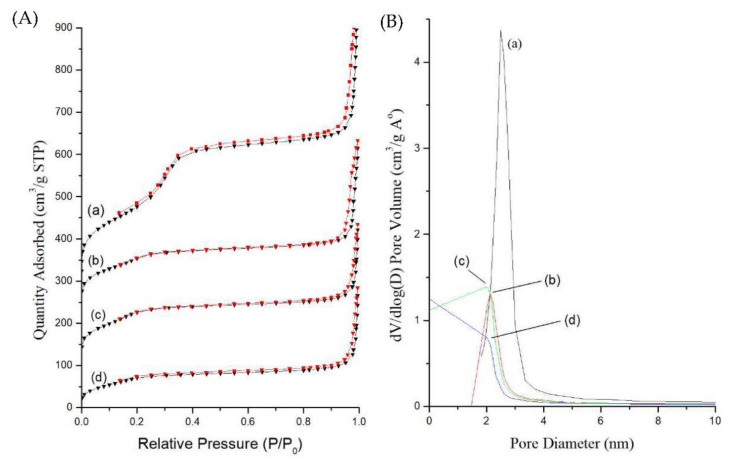
(**A**) Nitrogen adsorption (black line)–desorption (red line) isotherms and (**B**) pore size distribution plots of (a) MB-SiO_2_@MSNs (black line), (b) MB-SiO_2_@MSNs-IMD (red line), (c) MB-SiO_2_@MSNs-Cu (green line), and (d) MB-SiO_2_@MSNs-Cu-Dox (blue line).

Further, the textural properties of surface area, pore volume, and pore size of as-synthesized MB-SiO_2_@MSNs, surfactant extracted MSNs, silane modification, cupric coordination, and Dox loading were determined by nitrogen adsorption–desorption isotherms using the Brunauer–Emmett–Teller method (BET). As depicted in Figure 4A, the adsorption–desorption curve represents a type-IV isotherm with a small hysteresis loop. Due to the prominent condensation step at high relative pressure, the dried nanoparticles showed heavily agglomerated forms and nitrogen condensation in the interparticle voids. The adsorption isotherm of as-synthesized MB-SiO_2_@MSNs showed less surface area (256 m^2^/g) and pore volume (0.650 cm^3^/g), indicating that the nanochannels were blocked by CTA^+^ surfactants (Table 1). After extraction of CTA^+^ by NH_4_NO_3_/isopropanol solution, the surface area and pore volume drastically increased to 818 m^2^/g and 1.044 cm^3^/g, respectively. These results suggested the successful synthesis of MB-SiO_2_@MSNs with mesoporous structures and huge surface area. Furthermore, the surface-modified, cupric-coordination, and Dox-loaded samples showed a gradual decrease in their surface areas and pore volumes (MB-SiO_2_@MSNs-IMD: 386 m^2^/g, 0.592 cm^3^/g; MB-SiO_2_@MSNs-Cu: 380 m^2^/g, 0.486 cm^3^/g; and MB-SiO_2_@MSNs-Cu-Dox: 249 m^2^/g, 0.439 cm^3^/g) [48,49]. Considering these textural properties, it should be noted that the Cu-Dox complexes were well dispersed and immobilized inside the nanochannels of MSNs by imidazole silane. Specifically, the pore size analysis showed that the imidazole silane-modified MB-SiO_2_@MSNs samples were reduced from 2.5 to 2.1 nm. Subsequently, the loadings of Cu and Dox resulted in the further reduction of pore sizes of the designed nanoarchitectures (Figure 4B). It should be noted that the BJH method would underestimate the pore size distribution of the mesoporous samples [50]. However, in comparison between the samples, it could be concluded that the reduction in the surface area, pore volume, and pore diameter indicated the successful modification of MB-SiO_2_@MSNs and immobilization of Cu and Dox in the nanochannels of MSNs.

**Figure 5:**
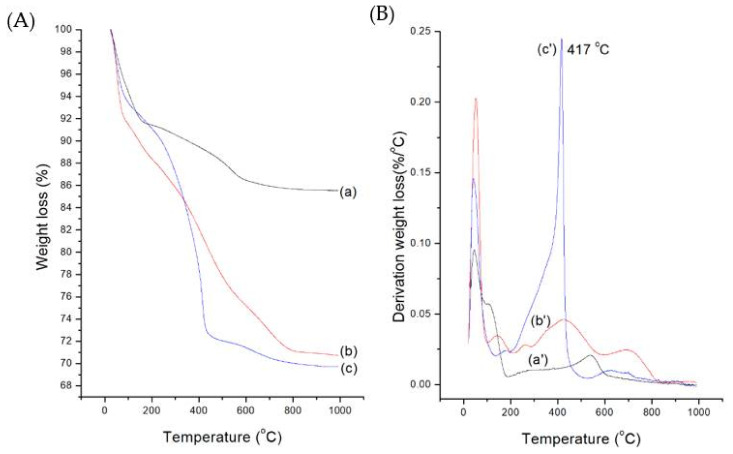
(**A**) TGA of weight loss profiles and (**B**) DTG profiles from the derivative weight loss of (a) MB-SiO_2_, (b) MB-SiO_2_@MSNs-IMD, and (c) MB-SiO_2_@MSNs-Cu-Dox.

Further, the successive immobilization of organic groups, including drugs, was confirmed by the thermogravimetric analysis (TGA) and derivative thermogravimetry (DTG) of MB-SiO_2_, MB-SiO_2_@MSNs-IMD, and MB-SiO_2_@MSNs-Cu-Dox. Figure 5A refers to the total weight loss curves, and Figure 5B denotes the derivation weight loss curves. The initial decomposition of all the samples in the heating process below 100 °C could be due to the loss of adsorbed water on the nanoparticle surfaces. Further, the weight loss after 100 °C could be due to the combustion of organic composites of imidazole silane, MB, and Cu-Dox in the MB-SiO_2_@MSNs-Cu-Dox. For the MB-SiO_2_@MSNs-IMD sample, the decomposition temperature of over 250 °C could be attributed to the weight loss due to imidazole groups. The MB-SiO_2_@MSNs-Cu-Dox in TGA studies showed an additional peak at 417 °C, which could be accumulated weight loss of organic modification and Dox degradation. The delayed degradation might have occurred due to chelation formation with quinolone moieties with Cu ions, respectively. The final loading amount of Dox was calculated as 7.5%, compared with bare MSNs (i.e., 2% wt of MSNs). Together, these findings indicated that the increased loading percentage of Dox was due to metal–ligand interactions with Cu (II) and Dox compared to Dox loaded in bare MSNs, which happened due to physical adsorption.

**Figure 6:**
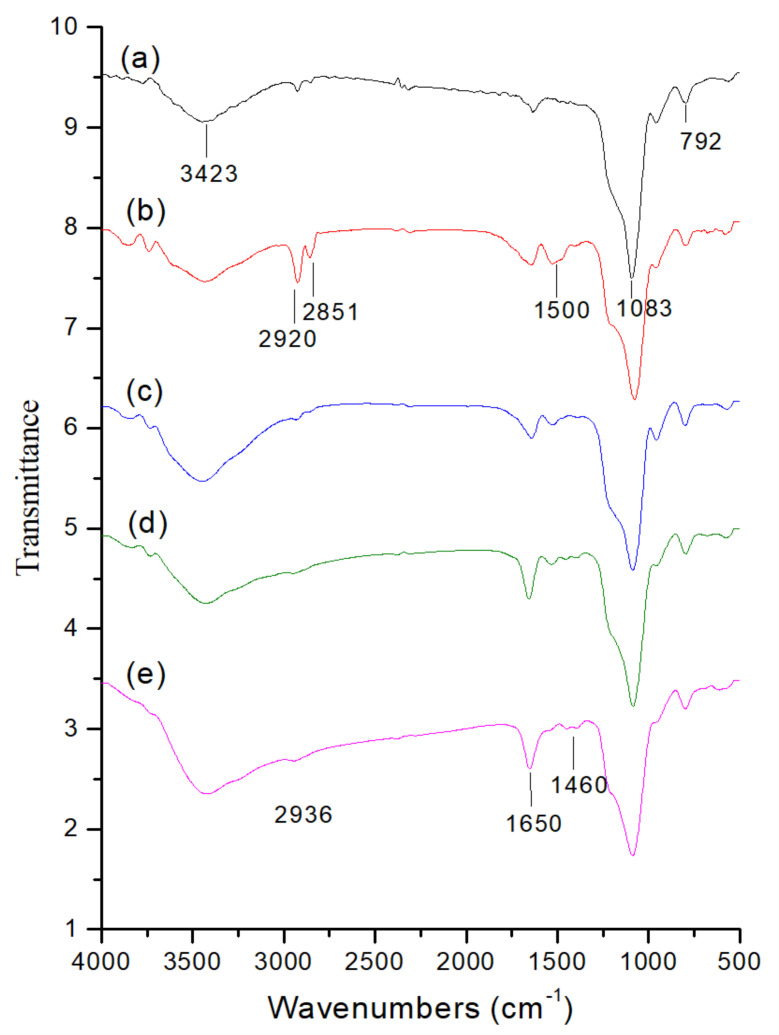
FT-IR spectra of (a) MB-SiO_2_, (b) MB-SiO_2_@MSNs-CTA^+^, (c) MB-SiO_2_@MSNs, (d) MB-SiO_2_@MSNs-IMD, and (e) MB-SiO_2_@MSNs-Cu-Dox.

Further, FT-IR spectroscopy was employed to explore the successful immobilization of chemical functionalities of the silica backbone and the organic groups in the designed core-shell nanocomposites (Figure 6). The broad spectrum at 3423 cm^−1^ could be ascribed to the O-H stretch of surface Si-OH and adsorbed water molecules (Figure 6a). The characteristic peaks at 1083 cm^−1^ and 792 cm^−1^ could be attributed to the Si-O-Si frameworks, resulting in the formation of stable silica structures. The high-intensity peaks at around 2851 to 2920 cm^−1^ and the C-H deformation peak at 1500 cm^−1^ could correspond to C-H stretching, indicating the CTA^+^ surfactant template of the MB-SiO_2_@MSNs-CTA^+^ sample (Figure 6b). After the surfactant was extracted in ammonium nitrate/isopropanol solution, the C-H stretching characteristic peaks were significantly reduced, indicating the successful extraction of the surfactant and resulting in the mesopores.

In the case of the Dox-loaded core-shell sample, the peak intensity at 1460, 1650, and 2936 cm^−1^ increased, which could be due to N-H, carbonyl, and C-H vibrational stretches of Dox, confirming its successful encapsulation in MB-SiO_2_@MSNs-Cu sample (Figure 6e) [51].

**Figure 7:**
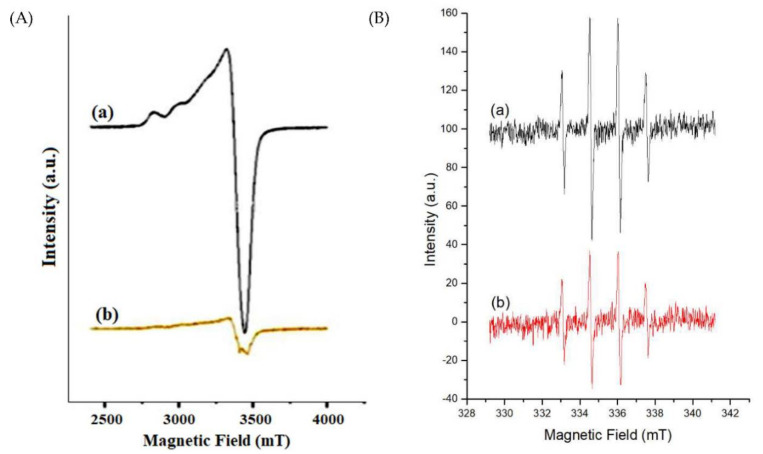
ESR spectra of (**A**) MB-SiO_2_@MSNs-Cu-Dox sample (a) before and (b) after release study in phosphate buffer (pH 5.0); (**B**) Spin trapping signal of **^˙^**DMPO-OH radical arising from (a) MB-SiO_2_@MSNs-Cu, and (b) MB-SiO_2_@MSNs-Cu-Dox sample reacted with H_2_O_2_.

Next, the ESR spectroscopy was applied to explain the characteristic feature of the Cu (II) coordination in the nanocomposites with 3d^9^ electronic spin (S = 1/2) and nuclear spin (I = 3/2). The spectrum of Figure 7A showed an anisotropic Cu(II) signal at 77 K. The calculated g values revealed that Cu(II) formation started sharing electrons in the nitrogen atoms of the imidazole ring and Dox quinone oxygen moieties. The g values, i.e., g_║_ (2.26) > g_┴_ (2.06), resulted due to the lone paired electrons’ characteristic spin in tetragonally elongated coordination (Figure 7A(a)). After a release study of the MB-SiO_2_@MSNs-Cu-Dox sample in phosphate-buffered saline (PBS, pH 5.0), we observed that the g value was near 2.0, due to the disassociation of pH-sensitive coordination bond expelling the Cu-Dox from the NPs (Figure 7A(b)). The PBS (pH 5.0)-soaked NPs had decreased Cu(II) signal, evidencing that Dox had a successfully triggered release by metal–ligand coordination [9].

Since Dox can trigger intracellular production of H_2_O_2_ levels, the mechanism for H_2_O_2_ production in CDT using the MB-SiO_2_@MSNs-Cu-Dox sample is represented in Equations (1)–(3). Dox is initially activated to a semiquinone radical in the mitochondrion (Equation (1)). Further, the oxygen species in the presence of semiquinone radicals reduce to superoxide by one-electron transfer (Equation (2)). Finally, the superoxide dismutation results in the generation of H_2_O_2_ (Equation (3)). In this study, the MB-SiO_2_@MSNs-Cu-Dox nanoparticles are internalized by cancer cells, triggering the pH-sensitive release of Dox in the acidic pH of endosomes. Thus, the released Dox can increase the intracellular H_2_O_2_ levels and catalyze the Cu(II) ions to produce cytotoxic hydroxyl radicals.

Dox → Dox semiquinone**^−^^˙^** (1)

Dox semiquinone^−**˙**^ + O_2_ → Dox + O_2_**^−^^˙^** (2)

O_2_**^−^^˙^**+ H**^+^** → H_2_O_2_ (3)

To further prove that Cu(II) ions in MB-SiO_2_@MSNs samples could catalyze a Fenton-like reaction (Equations (4) and (5)) to generate free radicals for CDT, we used a spin trapping technique to capture the active free radicals during the reaction (Equation (6)). The resultant hydroxyl radicals could be trapped by DMPO to form a **^˙^**DMPO-OH adduct as a stable radical. After the reaction, the liquid phase of extracted solution showed a signal of 1:2:2:1 hyperfine splitting pattern (a_N_ = a_Hβ_ = 1.496 mT). The signal intensities of **^˙^**DMPO-OH in MB-SiO_2_@MSNs-Cu and MB-SiO_2_@MSNs-Cu-Dox samples are displayed in Figure 7B. The EPR intensity showed that the MB-SiO_2_@MSNs-Cu sample displayed a high concentration of active Cu(II) ions in the Fenton-like reaction.

Cu^2+^ + H_2_O_2_ → Cu^+^ + H^+^ + HO_2_^˙^ (4)

Cu^+^ + H_2_O_2_ → Cu^2+^ + OH^−^ + **^˙^**OH (5)

DMPO + **^˙^**OH →**^˙^**DMPO-OH (6)

### 2.2. Cellular Uptake Studies

**Figure 8:**
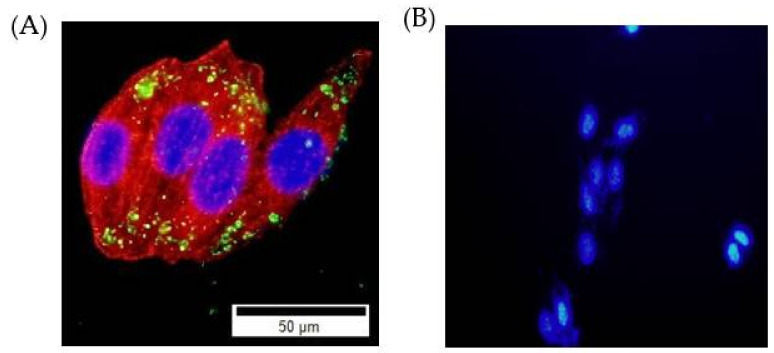
Fluorescent images to evaluate cell uptake of (**A**) FITC-labeled SiO_2_@MSN nanocomposites (green); cytoskeleton of rhodamine-phalloidin staining (red); DAPI nuclear staining (blue), and (**B**) MB-SiO_2_@MSNs-Cu-Dox-treated cells (with light) produced apoptotic bodies of condensed chromatin with bright fluorescence.

Typically, the nanoparticles enter cells through endocytosis pathways, the gateway into the cell [52,53]. It was observed that the designed FITC-conjugated MB-SiO_2_-MSNs showed high stability and cell uptake efficiency without leaching of MB. The NPs were internalized by the endocytosis mechanism, and a large number of nanoparticles were localized in the cytoplasm (Figure 8A). MB-SiO_2_@MSNs without Dox were non-toxic at high levels of nanoparticle concentration. Furthermore, achieving high Dox loading through Cu(II) metal affinity could trigger Dox release under the acidic pH of endosomes. The fluorescent images of DAPI staining after being treated with MB-SiO_2_@MSNs-Cu-Dox in B16 cancer cells successfully induced cell apoptosis, resulting in the change of nuclear morphology and chromatin condensation (Figure 8B).

### 2.3. ROS Studies

**Figure 9:**
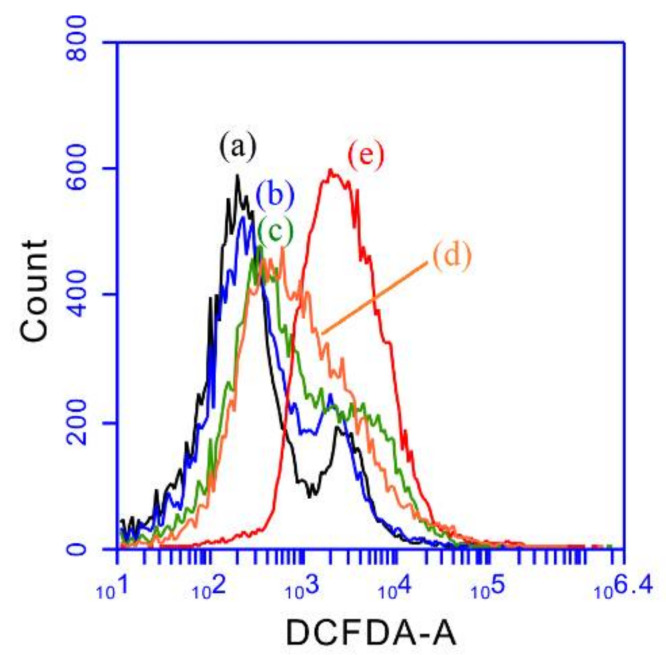
Evaluation of intracellular ROS by using DCFH-DA assay of (a) control (no nanoparticle treatment), (b) MB-SiO_2_@MSNs (dark), (c) MB-SiO_2_@MSNs (light), (d) MB-SiO_2_@MSNs-Cu-Dox (dark), and (e) MB-SiO_2_@MSNs-Cu-Dox (light) at the concentration of 50 μg/mL.

Further, we used a 2’,7’-dichlorofluorescein diacetate (DCFH-DA) probe to measure the intracellular reactive oxygen species (ROS) production. Typically, DCFH-DA enters the cells by passive diffusion, resulting in the cleavage of the two acetate groups of DCFH-DA and conversion into non-fluorescent DCFH by intracellular esterases. The oxidation of DCFH by various ROS (or H_2_O_2_) can generate highly fluorescent DCF, which shows excitation and emission at 485 nm and 535 nm. The treatments of MB-SiO_2_@MSNs and MB-SiO_2_@MSNs-Cu-Dox samples in the presence or absence of light irradiation were measured for ROS production by flow cytometric assay (Figure 9). Compared with the control group (no drug/nanoparticle treatments) and MB-SiO_2_@MSNs (dark) sample, the MB-SiO_2_@MSNs (light) sample showed a slight increase in ROS, mainly attributable to the photodynamic effects of MB. In addition, the MB-SiO_2_@MSNs-Cu-Dox (dark) treated cells showed a further increase in ROS levels. The maximum ROS production was observed under the light irradiation of the MB-SiO_2_@MSNs-Cu-Dox sample, indicating a combination of the synergistic effects from PDT effects of MB, chemotherapy of Dox, and chemodynamic therapy of copper ions. The ratios of mean fluorescence are 1: 1.2: 3.2: 2.6: 5.5 for the control, MB-SiO_2_@MSNs (dark), MB-SiO_2_@MSNs (light), MB-SiO_2_@MSNs-Cu-Dox (dark), and MB-SiO_2_@MSNs-Cu-Dox (light) sample. Thus, the ratios of ROS production contributed to from PDT only: chemotherapy, and CDT: PDT, chemotherapy, and CDT are 1.43:1:3.07. The results demonstrated that the combination of three synergistic effects could effectively produce the maximum levels of ROS and further induce DNA damage and cell death.

### 2.4. DNA Damage Studies

**Figure 10:**
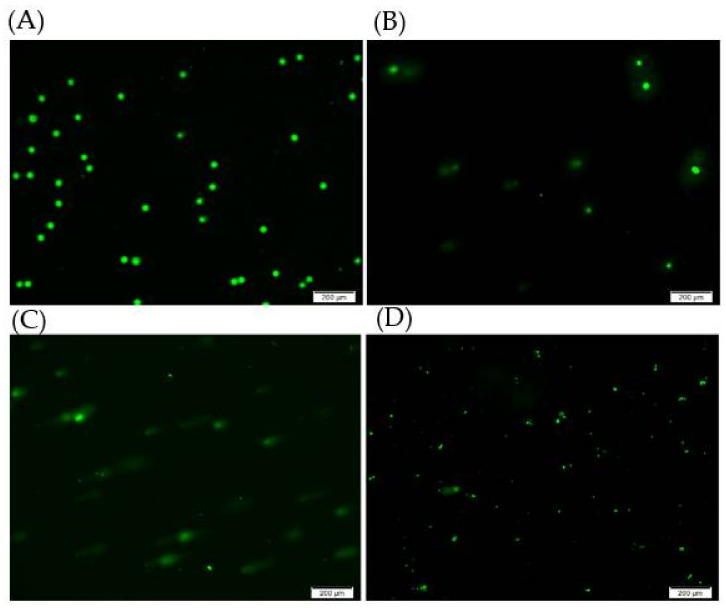
Comet assay from the single cell gel electrophoresis of B16 melanoma cells with (**A**) control (no nanoparticle treatment), (**B**) MB-SiO_2_@MSNs-IMD-Cu, (**C**) MB-SiO_2_@MSNs-Cu-Dox, and (**D**) MB-SiO_2_@MSNs-Cu-Dox (with light) at the concentration of 50 μg/mL.

Further, we used a comet assay of single cell gel electrophoresis to measure DNA damage from double (or single) strand breaks by nanoparticles. Our results showed that the MB-SiO_2_@MSNs-IMD-Cu sample induced a slight break for DNA with fewer signals for the comet tail (Figure 10). However, the MB-SiO_2_@MSNs-Cu-Dox-treated cells damaged most DNA, resulting in long-tailed comet signals. The mechanism lies in that the released Dox molecules could generate high levels of H_2_O_2_ concentration in cancer cells. Furthermore, Cu(II) ions could catalyze the H_2_O_2_ to produce highly toxic hydroxyl radicals and attack DNA for CDT. The MB-SiO_2_@MSNs-Cu-Dox sample was irradiated with light, generating the MB-assisted PDT efficacy and resulting in a large amount of singlet oxygen. Thus, the synergistic effect of PDT and CDT could trigger the heavy damage of DNA to break into smaller fragments. 

### 2.5. Cytotoxicity Studies

**Figure 11:**
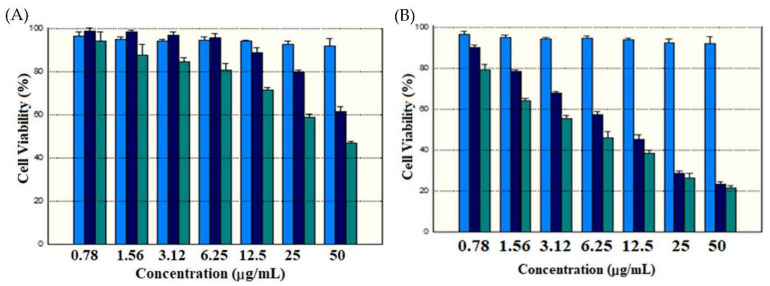
Cell viability by SRB assay of B16 melanoma cells after treatment of (**A**) MB-SiO_2_ (light blue), MB-SiO_2_@MSNs without light (dark blue), and MB-SiO_2_@MSNs with light (green) and (**B**) MB-SiO_2_ (light blue), MB-SiO_2_@MSNs-Cu-Dox without light (dark blue), and MB-SiO_2_@MSNs-Cu-Dox with light (green) at various nanoparticle concentrations.

Indeed, the potential toxicity of the carrier is always a great concern for nanomaterials used in drug delivery. To explore these aspects, we examined whether the delivery system would induce significant cytotoxicity in cancer cells under dark or light conditions (LED 630 nm, 15 mW/cm^2^ for 15 min). The cytotoxicity and anti-cancer activity of the designed nanocomposites were explored by incubation of various concentrations of nanoparticles for 24 h. As shown in Figure 11A, MB@SiO_2_ and MB-SiO_2_@MSNs samples without light irradiation showed low cytotoxicity. However, the MB-SiO_2_@MSNs-Cu-Dox-treated cells demonstrate a dose-dependent cytotoxic effect against B16 cells (Figure 11B). Further, the synergetic effect of nanocomposite with and without light irradiation was evaluated. The cell viability of MB-SiO_2_@MSNs (dark), MB-SiO_2_@MSNs (light), MB-SiO_2_@MSNs-Cu-Dox (dark), and MB-SiO_2_@MSNs-Cu-Dox (light) at 6.26 µg/mL treatment was 96%, 80%, 59%, and 47%, respectively. Therefore, the therapeutic effects from PDT only, chemotherapy plus CDT, and PDT plus chemotherapy plus CDT were in the ratios of 1:2.31:3.06. The combination therapy was proceeded by irradiation and further incubation for 20 h with MB-SiO_2_@MSNs-Cu-Dox nanocomposites. In the presence of a light source, MB absorbed the light kinetic energy and produced PDT to inhibit cell growth. Dox can perform chemotherapy and inhibit the progress of cell division. Moreover, the intracellular liberated Cu ions were involved in the Fenton-like reaction and produced free radicals to enhance the anti-cancer activity of CDT [54,55,56,57,58].

## 3. Materials and Methods

### 3.1. Materials

Cetyltrimethylammonium bromide (CTAB), fluorescein isothiocyanate (FITC), and tetraethoxysilane (TEOS) were obtained from Acros Organics Ltd. (Geel, Belgium). Potassium bromide was acquired from Fisher Scientific Ltd. (Loughborough, UK). Sulforhodamine B (SRB), ammonium nitrate (NH_4_NO_3_), N-(3-triethoxysilylpropyl)-4,5-dihydroimidazole, MB, and 2′,7′-dichlorofluorescin diacetate (DCFH-DA) were purchased from Sigma. Co. Ltd. (St. Louis, MO, USA). Copper sulfate was obtained from Showa Chemical Co. Ltd. (Gyoda, Japan). DMEM medium and Fetal bovine serum (FBS) were acquired from GIBCO/BRL Life Technologies (Grand Island, NE, USA). The comet assay kit was obtained from R&D systems (Minneapolis, MN, USA). Diamidino-2-phenylindole dihydrochloride (DAPI) and rhodamine-phalloidin were purchased from Invitrogen Ltd. (Eugene, OR, USA).

### 3.2. Characterizations

The micrometric surface area analyzer ASAP 2020 (Norcross, GA, USA) was used to determine N_2_-adsorption and desorption isotherms at 77 K for analyzing the textural properties. The surface area of various samples was obtained by the BET method. The pore size distribution curves were analyzed for the adsorption portion of the isotherms using the BJH (Barrett–Joyner–Halenda) method. The pore volume was obtained from the t-plot method. The chemical functional groups were confirmed by recording Fourier transform infrared (FT-IR) spectra on an Alpha spectrometer (Bruker, Billerica, MA, USA). Electron spin resonance (ESR) spectra were recorded using an EMX spectrometer (Bruker, Billerica, MA, USA) measured at 77 K. The physical composition of the nanoparticles was determined using thermogravimetric analysis (TGA) from the TA instruments. The surface morphology was determined by the transmission electron microscopy (TEM) images captured on a Hitachi H-7100 (Tokyo, Japan) operated at 100 kV.

### 3.3. Synthesis of MB@SiO_2_ Nanoparticles

Briefly, MB-incorporated non-porous silica nanoparticles as cores were synthesized using the previous procedure with slight modifications [59,60]. Initially, 14.5 mL of TEOS and 0.4 mL of MB (10 mg/mL of stock solution in deionized water) were added into a beaker containing 65.7 mL of ethanol (95%) and stirred for 20 min at 40 °C. Further, 4.9 mL of ammonia solution (28%) was added to the mixture and then stirred for another 6 h at the same temperature [61]. The MB-entrapped silica nanoparticles were collected by centrifugation and washed with ethanol. The nanoparticles were further dispersed in ethanol solution and stored at 4 °C. The product was named MB-SiO_2_.

### 3.4. Synthesis of MB-SiO_2_@MSNs

The mesoporous silica shell over the MB-SiO_2_ cores was grown using the conventional Stöber co-condensation method. Briefly, CTAB (300 mg) was completely dissolved in dd-H_2_O (60 mL). Then, 100 mg of MB@SiO_2_ was added to the above solution along with 1.1 mL of ammonia solution (28%), and the total mixture was stirred at a constant speed for 30 min at 40 °C. Further, the silica source, TEOS (0.5 mL), was added and stirring continued for another 6 h. The resultant core-shell solids were collected by centrifugation and washed twice with ethanol. Considering the templating mechanism, the CTAB molecules could be encapsulated as templates in cetyltrimethylammonium ion (CTA^+^). The product was shortly denoted as MB-SiO_2_@MSNs-CTA^+^. Finally, the template was removed via a highly efficient chemical extraction approach. The as-synthesized MB-SiO_2_@MSNs-CTA^+^ were dispersed in isopropanol (IPA, 50 mL) containing ammonium nitrate (NH_4_NO_3_, 30 mg) and continuously stirred for 24 h at 85 °C. Finally, the resultant product was collected by centrifugation (12,000 rpm for 17 min) and washed twice each with water and ethanol. The product was named as MB-SiO_2_@MSNs [62]. FITC-labeled MB-SiO_2_@MSNs were synthesized through the reaction of 3-aminopropyltriethoxysilane (APTES) with FITC molecules. Briefly, FITC (1 mg) and APTES 10 μL were dissolved in dried methanol (1 mL) and stirred at room temperature for 18 h in the dark. Further, FITC-labeled MB-SiO_2_@MSNs were synthesized by the same procedure of MB-SiO_2_@MSNs synthesis, except that the APTES-FITC conjugation was added to the TEOS beforehand. The co-condensation reaction proceeded in the dark.

### 3.5. Synthesis of Imidazole-Modified MB-SiO_2_@MSNs

Initially, 100 mg of MB-SiO_2_@MSNs was added to 80 mL of toluene and allowed to stir for 20 min. Then, 0.5 mL of N-(3-triethoxysulylpropyl)-4, 5-dihydroimidazole silane was added, and stirring continued for 6 h at 80 °C. The imidazole silane-modified nanoparticles were collected by centrifugation (12,000 rpm for 17 min) and washed with water and ethanol twice each. Finally, the product was denoted shortly as MB-SiO_2_@MSNs-IMD [63,64].

### 3.6. Coordination of Cupric Ions onto MB-SiO_2_@MSNs-IMD Nanoparticles

More often, the transition metals coordination is favorable by Lewis acid and base interactions between nitrogen and Cu atoms. Initially, 180 mg of copper sulfate as a Cu source was dissolved in 30 mL of dd-H_2_O at pH-6.0. Then, 200 mg of previously prepared MB-SiO_2_@MSNs-IMD was added and stirred for 30 min. The collected product by centrifugation (12,000 rpm for 17 min) was washed twice with ethanol and denoted as MB-SiO_2_@MSNs-Cu [65,66].

### 3.7. Immobilization of Dox onto MB-SiO_2_@MSNs-Cu

The anthraquinone functional group of Dox contains a lone pair of electrons, which could participate in a chelation reaction, resulting in the acidic pH-responsive coordination bond with metal ions. First, 5 mL of methanolic Dox solution at a concentration of 1.0 mg/mL was added with 100 mg of MB-SiO_2_@MSNs-Cu nanoparticles, and the pH of the mixture was adjusted to 6.0 and stirred for 24 h. The resultant solid was collected by centrifugation (12,000 rpm for 17 min) and washed twice with ethanol. The product was denoted as MB-SiO_2_@MSNs-Cu-Dox [67,68].

### 3.8. Cell Culture

The mouse melanoma cells (B16 cell line) were cultured in a DMEM medium with 10% FBS and 1% antibiotics (penicillin/streptomycin) maintained at 37 °C in 5% CO_2_.

### 3.9. SRB Assay

Initially, cells (1 × 10^5^ cells per well) were seeded in a 96-well plate and incubated overnight. Further, one control was fixed with trichloroacetic acid (TCA, 25 µL of 50% *w/v*) to arrest the cell cycle. Furthermore, the treatment groups and another control group were continued for further experiments. The treatment groups were added with different concentrations of nanoparticle samples dispersed in 100 µL of the medium. After 4 h of incubation, the respective treatment groups were subjected to the light treatment by using a LED 630 nm (15 mW/cm^2^) for 15 min and further incubation for 20 h. Later, 50 µL of cold TCA (50%) was added to other wells and incubated at 4 °C for 1 h. After decanting the medium and water washes, 100 µL of 0.4% (*w/v*) SRB solution (in 1% acetic acid) was added to all the wells and incubated for 20 min. The excess SRB was removed and further washed by using 1% (*v/v*) of acetic acid and subsequently solubilized by adding 10 mM of Trizma base (pH 10.5). Finally, the absorbance values were measured at 515 nm. Finally, the cell viability was calculated from the formula of [(Ctl − T_x_)/(Ctl)] × 100 [69].

### 3.10. Cellular Uptake Studies

Initially, cells at a density of 5 × 10^5^ cells were seeded in a 10 cm culture dish and incubated for 24 h for cell attachment. Then, 1 mL of FITC-conjugated nanoparticles (50 µg/mL) were dispersed in a medium and incubated for 24 h. Further, the culture medium was removed and washed twice with PBS. Then, a series of steps were followed to prepare the cells for immunostaining of fluorescent imaging, formalin (3.7%, 1 mL, 10 min) for cell fixation, Triton X-100 (0.1% in PBS, 1 mL, 5 min) for cell membrane permeation, BSA (3%), rhodamine-phalloidin (1 µg/mL, 30 min) for staining cytoskeleton, and DAPI (2 µg/mL, 5 min) for counterstaining nucleus. Finally, the cells were visualized under fluorescence microscopy [70,71].

### 3.11. Comet Assay

Single-cell gel electrophoresis of the comet assay can use to evaluate the DNA damage after treatment with MB-SiO_2_@MSNs-IMD-Cu and MB-SiO_2_@MSNs-Cu-Dox nanoparticles (50 μg/mL). The treated cells (1 × 10^5^/mL, 5 µL) were mixed with 50 μL of Comet low-melting agarose and then added to 50 µL of the above mixtures and placed onto CometSlide^TM^. Slides were immersed in 4 °C lysis solution (60 min), drained excess buffer, and then immersed in alkaline unwinding solution (4 °C for 1 h). Further, slides were placed in the alkaline electrophoresis solution to perform at 21 V, 300 mA for 20 min. DNA was stained by adding 100 µL of Sybr green (1:10,000 dilution) for 30 min. The DNA damage was measured by fluorescent microscope (Ex/Em: 496/522 nm).

### 3.12. DCFH-DA Assay

Briefly, a DCFH-DA fluorescent probe was applied to detect the production of intracellular ROS levels. The cells were cultured in a 6-well plate at a density of 2 × 10^5^ cells/well for 24 h. After appropriate cell attachment, various nanoparticles (50 μg/mL) were added to the culture medium and incubated for 4 h. Cells were further washed with PBS twice and 10 μM DCFH-DA was added in each well and incubated for 30 min. The cells were then washed with PBS, and PDT groups were further exposed to the LED 630 nm (15 mW/cm^2^) for 15 min and then incubated for 12 h. Finally, the cells were analyzed by flow cytometry to determine the fluorescent intensity.

### 3.13. ESR Spin Trapping

To explore the free radical production of MB-SiO_2_@MSNs-Cu and MB-SiO_2_@MSNs-Cu-Dox samples through the cupric catalysis of Fenton-like reaction with H_2_O_2_, the ESR spin-trapping technique was studied by using DMPO as a free radical trapping agent. The experimental conditions for a typical reaction included 6 mg of nanoparticles, 50 µL of 100 mM DMPO, 50 µL of 50 mM H_2_O_2_, and H_2_O (400 µL). After vigorous vortexing of the mixture for 20 s, the solids were removed by syringe filters. The ESR spectra of the extracted liquid were recorded after mixing for 3 min.

## 4. Conclusions

The MB-SiO_2_ embedded mesoporous silica core-shell architectures were designed by initially incorporating MB in silica frameworks and further depositing mesoporous silica shells. Further, the surfactant template was successfully extracted and modified with imidazole silane to achieve Cu ion chelation and high loading of Dox in the nanochannels of MSNs. This material possesses large advantages in cancer studies, good controllable size and surface charge, and carries more than one drug at a time to inhibit tumor growth synergistically. These designed architectures were systematically characterized using various physicochemical characterization techniques and demonstrated the potent anti-cancer efficacy against skin melanoma. Together, our results demonstrated that the mesoporous silica-based core-shell nanoarchitectures shows great potential as an effective strategy in synergistically ablating cancer through chemo-, chemodynamic, and photodynamic therapies.

## Figures and Tables

**Table 1:** Textural properties of the various nanocomposites.

Sample	Surface Area ^a^ (m^2^ g^−1^)	Pore Volume ^b^ (cm^3^ g^−1^)	Pore Diameter ^c^ (nm)
MB-SiO_2_@MSNs-CTA^+^	256	0.650	N.D. ^d^
MB-SiO_2_@MSNs	818	1.044	2.5
MB-SiO_2_@MSNs-IMD	386	0.592	2.1
MB-SiO_2_@MSNs-Cu	380	0.486	N.D. ^d^
MB-SiO_2_@MSNs-Cu-Dox	249	0.439	N.D. ^d^

^a^ Determined by the BET method. ^b^ Determined by the t-plot method. ^c^ Determined by the BJH method in the adsorption branch of the isotherm. ^d^ N.D. = Not determined.

## Data Availability

Not applicable.
